# Early-life environmental enrichment promotes positive animal welfare for juvenile Atlantic salmon (*Salmo salar*) in aquaculture research

**DOI:** 10.1038/s41598-025-88780-0

**Published:** 2025-02-18

**Authors:** Pamela M. Prentice, Mauro Chivite Alcalde, Petr Císař, Sonia Rey Planellas

**Affiliations:** 1 Institute of Aquaculture, Faculty of Natural Sciences, University of Stirling, Stirling, UK; 2 University of South Bohemia in České Budějovice, FFPW, CENAKVA, Zámek 136, 373 33 Nové Hrady, Czech Republic; 3 Centro de Investigación Mariña, Laboratorio de Fisioloxía Animal, Departamento de Bioloxía Funcional E Ciencias da Saúde, Facultade de Bioloxía, Universidade de Vigo, Vigo, Spain; 4 Present Address: Animal Behaviour and Welfare, Animal and Veterinary Science Research Group, SRUC, West Mains Rd, Edinburgh, EH9 3JG UK

**Keywords:** Fish welfare, Structural enrichment, Social behaviour, Cognitive abilities, Neurogenesis, Animal behaviour, Animal physiology

## Abstract

Early life experiences have long-lasting effects on behaviour and physiology, influencing development of adaptive natural behaviours. Enriching farmed environments encourages expression of natural behaviours in captive fish, promoting positive animal welfare, important for conducting valid and reproducible research and informing better management practices. Using juvenile Atlantic Salmon (*Salmo salar*), we tested whether provision of environmental enrichment in early life improves welfare. Welfare indicators were measured comparing enriched to non-enriched tanks. Morphological (fin damage and body condition), physiological (plasma cortisol) and behavioural traits (activity, group cohesion, and neophobia) were recorded. Molecular expression of brain mRNA transcripts related to stress response, neuroplasticity and serotonergic system was analysed. Environmental enrichment did not affect morphological welfare indicators, activity, or cortisol. Enriched fish were more cohesive than non-enriched fish, less neophobic, with higher serotonergic turnover, suggesting enrichment mitigates against stress, promoting positive emotional states. Genes related to neuronal development and activity (*bdnf* and *ndf1*), cellular stress (*hsp90* and *hsp70*), and serotonin synthesis *(tph2*) increased in enriched fish following stress, enhancing cognitive function. Our findings suggest early life environmental enrichment is advantageous for positive animal welfare by improving emotional states in captive environments, ensuring animals are free of negative experiences and able to access positive ones.

## Introduction

Aquaculture has experienced rapid growth in the number of farmed fish species used commercially^1^. Similarly, there has been a significant increase in the use of fish within scientific research for aquaculture purposes^2–4^. However, compared to their terrestrial counterparts, the welfare of fish has often been overlooked, raising concerns within the public and the scientific community^5^. The increase in attention has stemmed from a central debate around whether fish are sentient or not^6^. Besides its controversial nature, recent evidence strongly suggests fish can feel pain, display high cognitive abilities, engage in complex social interactions, and experience positive and negative emotional states^3,6^.

Ensuring optimal fish welfare is not only a legal and ethical responsibility, but it is also fundamental for conducting valid and reproducible research. Impaired animal welfare can lead to physiological and behavioural changes that may significantly impact the accuracy of scientific findings^3^. To achieve optimal welfare, fish should be housed in appropriate conditions that encompass the 5 domains (nutrition, environment, health, behaviour and mental states), all of which should be considered when assessing welfare^7^. In line with the 5 Freedoms Act^8^, animals should be free from hunger and thirst; discomfort; pain, injury and disease; fear and distress; and be free to express natural behaviour. This concept was expanded in the 5 domains model by Mellor (2016) to not only include suffering experienced from negative states, but to focus on mental state and positive aspects that improve animal welfare^7^.

Assessing animal welfare is a multifactorial task and requires a holistic approach measuring a variety of factors. These include aspects such as body integrity, physiology, behaviour and mental well-being, among others. In the case of salmon, specific welfare indicators have been developed that provide valuable information to accurately assess the status of the fish^9^. Traditional welfare interventions focus on minimising negative physical and physiological welfare states (e.g., pain, hunger, disease and stress). Significantly less focus (if any) is given to promoting positive welfare states, such as the expression of rewarding behaviours (e.g. exploration, foraging, social interactions), which can elicit positive effective sates^10^ referred to as Positive Animal Welfare (PAW). PAW has recently been defined as flourishment of an animal experiencing positive mental states, leading to the development of competence and resilience^11^. Thus, PAW goes beyond just ensuring optimal health and the prevention or alleviation of suffering, by further offering rewarding experiences so that animals may thrive in captivity.

One way to promote PAW is through enriched housing that mimics wild, more complex habitats, encouraging the expression of natural behaviours^12^. Environmental enrichment (EE) includes the addition of structures or stimuli that elicit and engage the fish’s response to mental and physical challenges (e.g., shelters, novel substrates, variation in water velocity or temperature;^13^). The addition of EE within aquaculture rearing tanks (which are generally homogenous environments devoid of stimulation), has gained increasing recognition as a promising approach to mitigate against the adverse effects of captivity on fish welfare (e.g. opportunities to escape dominant conspecifics, stress induced by overcrowding or transportation^14^). Housing conditions with enhanced complexity and novelty have been shown to reduce stress, improve behavioural diversity including promoting natural behaviours, and enhance cognitive function, promoting positive welfare states in fish^15,16^.

It is important to consider life-stage specific EE to meet the behavioural and physiological needs of distinct captive species. Wild juvenile salmonids (a family of widely cultured fish, including salmon and trout), inhabit rivers and coastal areas comprising rocks, vegetation, and variable water velocities^17^. They have a diverse behavioural repertoire during early life that includes exploration, foraging, and social behaviours. The development of these behaviours is essential for their well-being. Early life experiences impact phenotypic expression of behaviour in later life, including coping strategies and environmental adaptability^18^. For example, juvenile three-spined sticklebacks (*Gasterosteus aculeatus*) develop anti-predator responses through social learning in early life, and this behaviour is more developed in populations under high predation^19^. In juvenile Atlantic salmon, early life exposure to enriched conditions increases boldness^20^, and improves cognition and memory^21^. Conversely, barren homogeneous environments in captivity can deprive fish of crucial experiences, and animals may develop maladaptive behavioural traits and abnormal cognitive abilities that negatively affect welfare^22^. For example, juvenile salmon reared in barren hatchery conditions have higher mortality with lower reproductive success in adulthood, compared to those reared in natural streams^23^. Providing EE that mimics natural environments during early-life stages may promote natural programming of adaptive phenotypes both behaviourally and physiologically.

In addition to encouraging natural behaviours, EE has been shown to promote cognitive function in fish^24^. For example, adding complexity (structural enrichment) to the rearing environment in juvenile Atlantic salmon (*Salmo salar*), improved brain plasticity and cognitive performance in a spatial learning task^21^. Most rearing environments are barren and lack cognitive stimulation, which can increase depressive like states in mammals (e.g. anhedonia; for a review see^25^), thereby compromising welfare^26^. The provision of EE can increase cognitive stimulation and behavioural flexibility in response to changing environmental conditions^22,27,28^, enabling individuals to cope more effectively with stressful situations^29,30^. The neuronal proliferation associated with behavioural flexibility is important in terms of animal welfare^31^. For example, neurotransmitters such as serotonin (5-HT), play a key role in the control of cognitive and behavioural processes such as, but not limited to, anxiety, depression, aggression, and the stress response^32^. The provision of environmental enrichment has been shown to change brain monoamine levels in some aquaculture species^28,33,34^. Importantly, 5-HT is critical in controlling neurogenesis and synaptic plasticity, processes that are crucial in learning, memory, and affective state, and which consequently deteriorate rapidly when the serotonergic system malfunctions^35–37^.

Here, we investigate the effects of structural EE on morphological, behavioural and physiological welfare indicators in juvenile Atlantic salmon (*S. salar*). We reared early life stages of Atlantic salmon fry from first feeding to 13 weeks in structurally EE, or non-enriched (NE) control tanks. We scored individual fish before and after rearing in treatment tanks using a morphological welfare scoring scheme commonly used in aquaculture (e.g. fin damage, body condition, weight and length^9^). Fin damage is a universal indicator of aggression in salmon^38^, and the presence of EE can affect levels of aggression^39^. EE has been shown to reduce aggression in salmonid hatcheries (measured as reduced dorsal fin damage; for a review see^40^), by potentially increasing shelter availability and opportunities to evade dominant conspecifics. However, the effect of EE is likely to be species specific, and dependent on EE complexity and construct. For example, if the number or volume of EE is too low in relation to fish density and tank size, EE can become a defendable resource, increasing aggressive interactions due to resource guarding^40^. Further morphological indicators commonly measured in aquaculture are weight, length, and body condition^9^, and are important in terms of health and productivity. Although EE can increase aggression in some species^41^, resulting in reduced growth and survival, evidence suggest EE has no effect on early life rearing performance in salmonids^42,43^. It is therefore essential to verify the effects of different types of EE on multiple welfare indicators across species and life stages.

Behavioural welfare indicators in salmon were also recorded throughout the experiment, including group cohesion and activity^9,44^. In the wild, salmonids exhibit both shoaling and solitary behaviour, displaying lower group cohesion (more dispersed group behaviour) under stable environmental conditions^45^. In captive environments, in the absence of threatening stimuli, fish are expected to be more dispersed, utilising and exploring all the available space. In the presence of EE, shoal cohesion can increase, as observed in rainbow trout (*Oncorhynchus mykiss*)^46^. In this study fish in enriched housing were more cohesive but less aggressive, indicating positive welfare states potentially driven by enhanced group stability in this species^46^. It remains unclear whether increased cohesion consistently reflects positive welfare states, as the direction of the effect may depend on contextual factors such as species-specific behaviours, environmental conditions, and the definitions used to assess cohesion.

Indeed, definitions of cohesion vary across behavioural studies and can influence the interpretation and direction of observed effects. Cohesion is commonly quantified as the distance between individuals and may include swimming polarity (where fish orient themselves in the same direction). Synchronised and polarised swimming, combined with close proximity, is referred to as schooling^45^. Fish may increase schooling behaviour when threatened^47^ which is generally associated with heightened arousal, stress, and negative welfare states (especially in captive or threatening conditions)^48^. This contrasts with shoaling behaviour, where fish remain together for social purposes without necessarily swimming synchronously or exhibiting polarity^45,48^. In this study, cohesion is defined as the mean neighbour distance between fish^49^, without requiring polarised swimming. Under this definition, cohesive behaviour ranges from tight schooling among neighbours, often indicative of heightened arousal and a defensive state, to more dispersed behaviour, which may reflect lower arousal and a more relaxed or exploratory state, where fish are spread out in the tank.

Activity is another commonly used indicator of welfare states in fish. Changes in activity can reflect how fish perceive and respond to the surrounding environment^44^. Increased activity correlates with increased feeding anticipatory behaviour, which can reflect positive welfare states^44,50^. Similarly, heightened activity is often associated with increased exploratory behaviour in fish,^51^ which also suggests positive welfare^51^. Conversely, an increase in activity of accelerated burst reactions or abrupt changes in swimming trajectories—as observed in escape responses^52^—can indicate stress or fear, reflecting negative welfare states^53^. Changes in activity within a normal range can also represent regular swimming behaviour and do not necessarily indicate stress^54^. Therefore, caution is advised when interpreting activity levels, and clear definitions of the observed trait are essential. Activity should always be considered alongside other measures to determine the direction of its effect. For example, Atlantic salmon exhibit elevated swimming activity following stressful husbandry practices and veterinary treatments, in addition to elevated heart rates^51–53^. In the present study, activity is measured as the mean distance swum by all fish within each frame of video clips (detailed in the methodology). We note that changes in activity here could reflect stress or fear responses but may also indicate exploratory or foraging behaviour. These changes are interpreted in conjunction with other behavioural, physiological, and morphological traits to provide a comprehensive assessment.

We further tested the effects of EE on behaviour within a novel object test, a common paradigm used to quantify ‘neophobia’, fear or anxiety in response to novelty^55^. Latency to resume exploration (or normal activity) following exposure to novelty is a common behavioural proxy for neophobia^56,57^, and can provide insight into stress coping in captive animals^58,59^. EE can increase exploration^20,33^, and can promote recovery from stress^40,60,61^. If enrichment is important for salmon welfare, we predict that fish housed with EE will be more dispersed (reflecting lower states of arousal), show reduced swimming activity (reflecting increased exploratory profiles), and display less fearfulness in the novel object test compared to NE fish.

At the end of the experimental period, fish were exposed to a stressor (chasing with a net), following which physiological welfare indicators were measured. These included plasma cortisol, brain monoamines, in addition to multiple molecular parameters indicative of cognitive capacity, neuronal activity, stress response, and the activity of the serotonergic system. Previous studies in salmonids have shown that the serotonergic system is activated in response to stressful stimuli, in line with an activation of the endocrine stress response. This activation could imply an integrative role of the serotonergic system at the central level, acting as a tonic modulator of the HPI axis^62,63^. EE can impact fish cognitive abilities, involving an increased ability to perceive the surrounding environment^64^. In this study we also aim to assess whether EE affects serotonergic activity following exposure of an acute stressor, and thus whether an increased cognitive capacity results in a differential stress axis response at the level of the serotonergic system and/or the HPI axis. In this study, we present a complete multifactorial approach to determine the role of EE on the positive welfare of fish used in research for farming purposes. Our objective is to improve the emotional state of the fish in captive environments and ensure animals are not only free from negative experiences and are also able to access positive ones. By increasing their general welfare, we expect to improve experimental reliability, replicability, and reproducibility, one of the major challenges we presently face in science.

## Methods

### Ethics

This work was conducted under the auspices of the UK Animals (Scientific Procedures) Act (1986) with approval of the University of Stirling research ethics committee (AWERB 2022 7013 5863). Experimental procedures and behavioural assays were developed in accordance with the principles of the three Rs and ASAB guidelines^65^, and written up following ARRIVE guidelines^66^. As such, animals housed in control tanks were returned to the stock population for reuse in later trials. All periods of handling and emersion were kept to a minimum and only fish deemed healthy and exhibiting normal behaviour were used in trials. End points were considered at each stage of the experimental protocol.

### Experimental fish and treatment

Fish used in this study were Atlantic salmon fry (*S. salar*) hatched and raised at the University of Stirling’s Niall Bromage Freshwater Research Unit (NBFRU). All data was collected between August and December 2022. After resorption of their yolk sack, fry (n = 3000, 1 g in weight) were randomly distributed equally among eight identical Recirculatory Aquaculture System (RAS) tanks (n = 375 per tank). RAS tanks were 1 m in diameter (0.78 m^2^) with a total volume of 700 l, and a water renewal rate of 900 L/h. The water temperature was maintained at 12 °C and tanks were covered with lids with an integrated light (providing 24 h light). A SWAN CCTV camera was mounted onto each of the RAS tank lids for behavioural recording. Each tank was equipped with an automatic Arvo-Tec feeder that provided continuous ad libitum commercial fish feed from BioMar Ltd. Husbandry practices included daily mortality checks and cleaning of the central outlet pipe, further to weekly tank cleaning (including the enrichment structure) and sampling to check weights. All fish were kept under these conditions for the duration of the experiment (13 weeks).

**Fig. 1:**
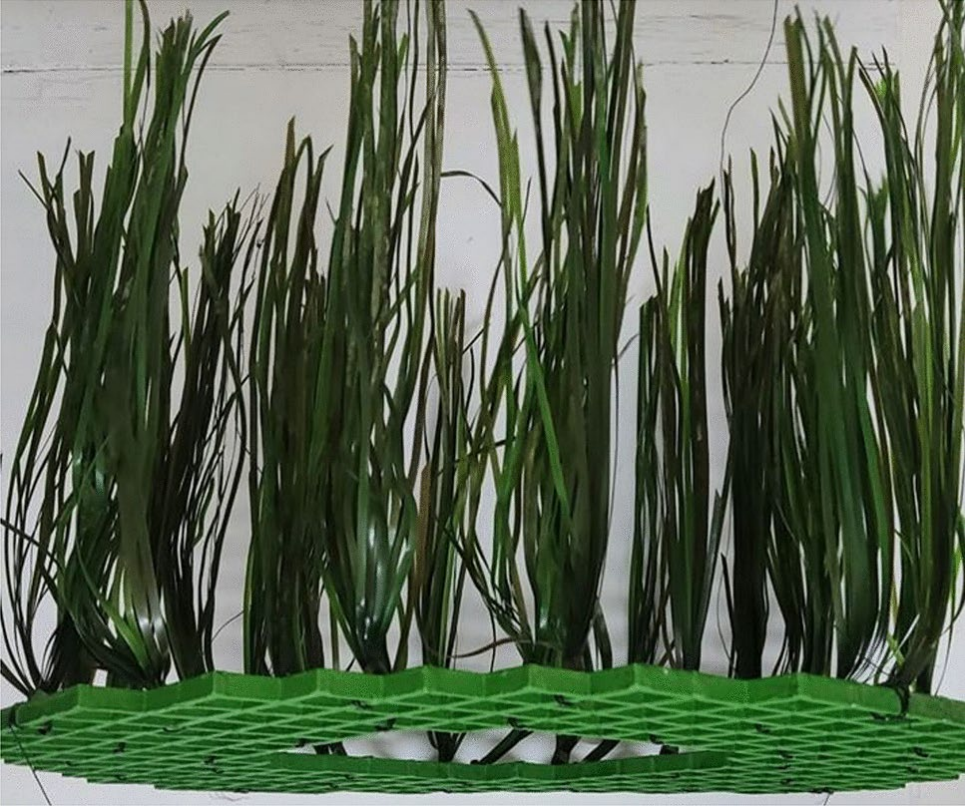
Example of the physical EE structure, showing PVC grid from which artificial plants are suspended.

Prior to the start of the experiment, fry was left to acclimate in the RAS tanks for 14 days. Following which, structural EE was added to four randomly assigned RAS tanks, resulting in four EE tanks (4 × EE), and four NE barren tanks (4 × NE). The enrichment consisted of a smooth circular PVC grid (70 cm diameter) covered with artificial plastic plants (www.pangearocks.com). Each plant (n = 28) consisted of 12 individual seagrass leaves (60 cm length) grouped together and attached to the PVC grid (Fig. 1). The enrichment structure was suspended from the rim of the tanks, providing cover throughout the water column created from vertical suspension of seagrass leaves.

### Measuring Welfare Indicators

**Fig. 2:**
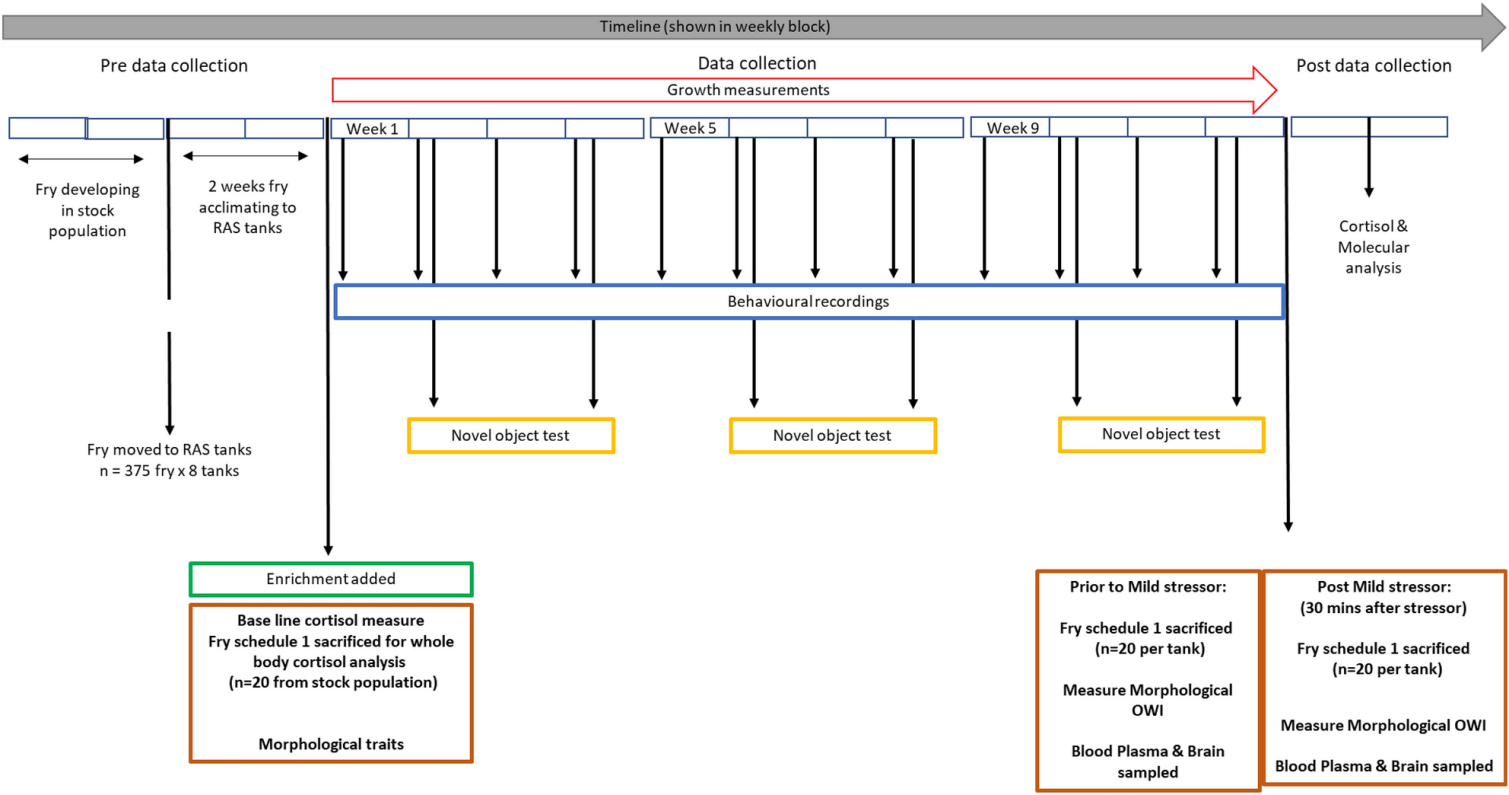
Timeline of the experimental procedure showing periods of data collection.

To assess the impact of EE on juvenile salmon, we measured morphological, behavioural, and physiological welfare indicators throughout the experiment, comparing EE with NE treatment groups. An overview of the experimental timeline can be seen in Fig. 2.

- Morphological

**Fig. 3:**
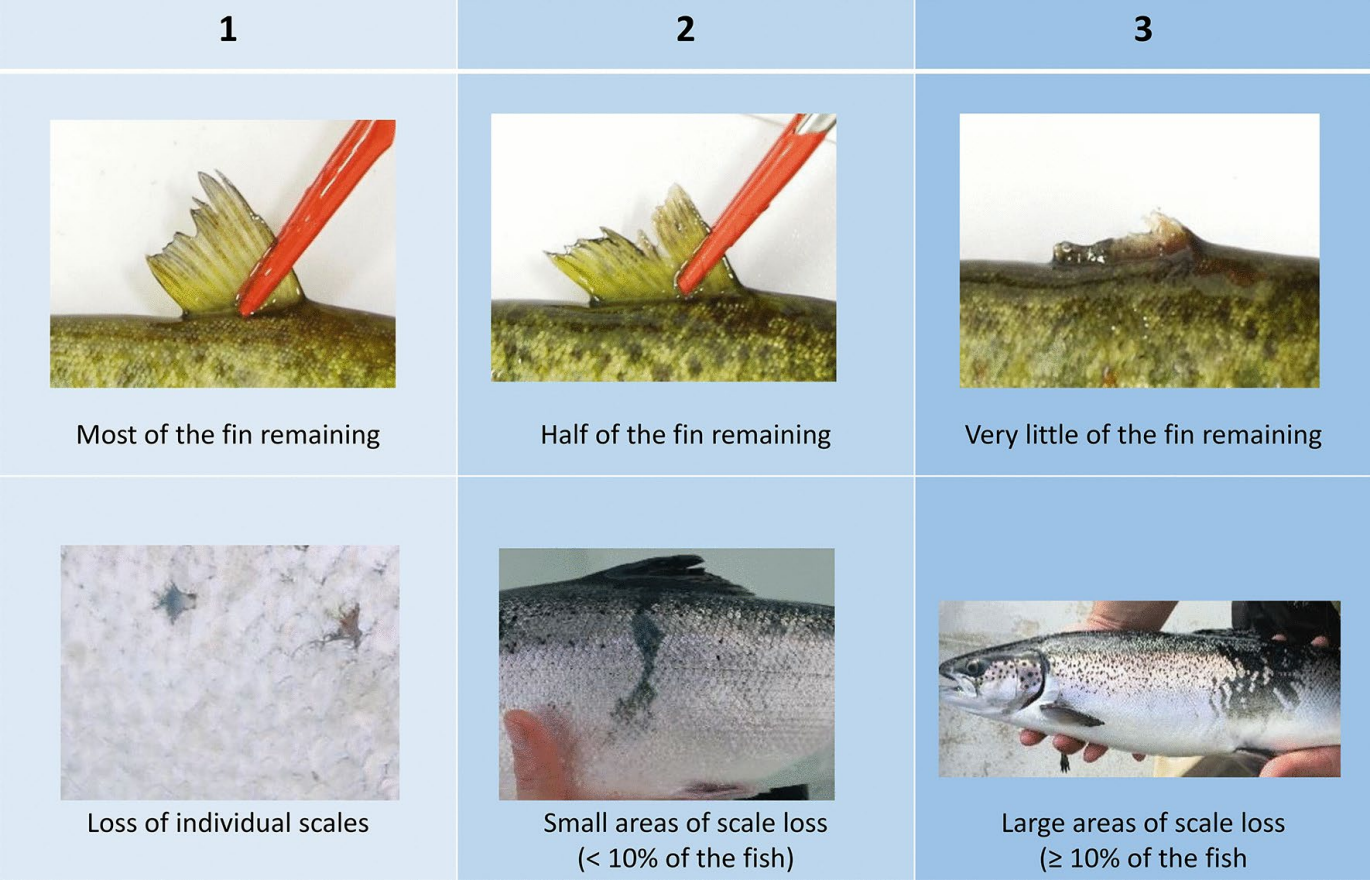
Morphological welfare indicator scoring scheme for classifying key external injuries. Shown here are dorsal fin damage and body condition scores across 3 levels. Level 0: Normal condition, Level 1: Minor occurrences, Level 2: Compromised condition. Copyright Noble et al., (2018).

To assess whether EE affects morphological welfare indicators, we compared morphological scores from samples of fish at the beginning, with scores at the end of the experimental procedure. Fish were sampled (n = 20 fish per tank) at week 0, and again at the end of the experiment (pooled across two sampling points: n = 10; week 12, n = 10; week 13). Experimenters were blind to treatment where possible. The sample size was determined in accordance with the UK National Centre for the 3Rs (NC3Rs) and the ARRIVE guidelines^65,66^, aiming to minimise the number of animals used while ensuring the generation of robust and reliable results (n = 20 fish per tank, ~ 5.3% of the population per tank). Fish were randomly selected to ensure representativeness and minimise selection bias. During each sampling session, fish were weighed, and length measured, and the following welfare indicators (WI) scored; dorsal fin damage, and body condition. The indicators were measured using the FISHWELL scoring scheme^9^ and scored on a scale of 1–3 (Level 1: Normal condition, Level 1: Minor occurrences, Level 3: Compromised condition; see Fig. 3 for details).

- Behavioural

**Fig. 4:**
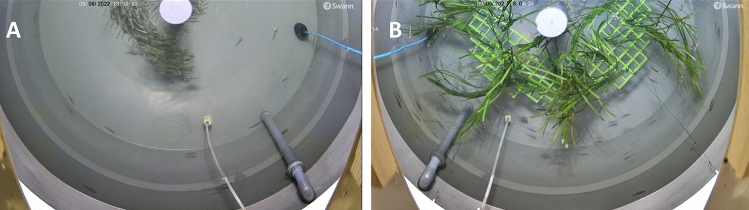
Overhead camera view of (**A**) non-enriched, and (**B**) an enriched tank, showing PVC grid with artificial plants suspended in the water column.

Behaviour was recorded throughout the experimental period with an overhead camera attached to the tank lids, oriented towards the bottom of the tanks, capturing the lower half of each tank’s field of vision (see Fig. 4). While this setup limited data collection, it was necessary due to equipment constraints imposed by RAS tank lids and camera dimensions. There was potential for bias with this setup, however preliminary observations did not reveal significant behavioural localisation. The recorded section was standardised across all tanks, ensuring consistent environmental conditions (e.g., lighting, temperature, flow) and minimising potential gradients that could affect fish behaviour (including food distribution, and shelter from outflow pipes, etc.). Video recordings (automated to reduce disturbance) captured footage for 30 min at 4 timepoints each day (6 am, 12 pm, 6 pm and 12 am), aided by a 24-light cycle.

To extract quantitative data of fish behaviour we used inhouse software to analyse the video footage. The software was implemented in MATLAB, and based on statistical modelling using the tank background to represent the monitored scene and background subtraction to detect the fish group, fish individuals and enrichment structure (following^67^). The software produces the position and size of each fish group, positions of individuals, and area of the enrichment for all consecutive video frames. To compare the behaviour of salmon fry under EE with NE conditions, we analysed the following traits: *group cohesion*, a*ctivity*, and *enrichment occupation,* calculated from the parameters produce by the software (trait definitions are outlined in Table 1).

**Table 1:** Ethogram of behavioural welfare indicators.

Behaviour	Description	References
Overhead Camera Behaviours		
Group cohesion	Defined as the mean neighbour distance between fish within the main group (minus any outliers), in each tank. Swimming polarity is not considered Calculated as the ratio between area of the tank occupied by the main group, and the total area of the tank monitored by the camera. The mean ratio across all video clips was measured for each tank Categorical score: 0 = Tight; ratio < 0.25 1 = Loose; 0.25 > ratio < 0.75 2 = Dispersed; ratio > 0.75	^46,67,69^
Activity	Mean distance swum by all fish (cm) Calculated as the mean movement of fish, based on differences in detected positions between consecutive frames. Fish are identified as part of a group (detected as a single object due to their proximity) or as individual objects if not connected to the group. The centroid (centre of mass) of each detected object is tracked, and the Euclidean distance between centroids in consecutive frames is calculated. These distances, representing the movement of each object, are weighted by the size of the object (normalised to the number of fish within it). The mean of these distances across all objects and frames is used as the measure of overall activity	^46,67,70^
Enrichment Occupation	Percentage of fish under the enrichment (%) Calculated as the ratio between area occupied by fish under the enrichment, and the total area occupied by all fish	^67^
Novel Object Test		
Latency to resume normal activity	Time taken for fish to resume “normal swimming” (seconds) Normal swimming resumed when the occurrence of darting behaviour (sudden, rapid, high velocity movements) was less than 2 fish per 10 s	^71–73^

Group cohesion and enrichment occupation = parameters are corrected to account for fish growth over time. Normalisation of fish size is based on the heuristic rule, to account for fish occupying a larger area of the tank over time. See^67^ for full details.

To further assess the effect of EE on behaviour, we exposed each tank to a novel object test, once every 2 weeks (6 trials per tank in total). During which, the experimenter submerged a Go Pro camera attached to the end of metal pole (2 m) into each tank and recorded the response of fish to the novel object for 30 min. From this footage the trait *latency to resume normal activity* (see Table 1 for a definition) was extracted and quantified using the software BORIS^68^. It was not feasible to fully blind data collection as EE was visible in the tanks, however observers were blind to tank numbers to minimize bias as much as possible.

- Physiological

To assess the effects of EE on stress physiology and neuroplasticity, we measured multiple physiological WI across the 3 sampling sessions. Firstly, we measured a population base line cortisol level by sampling fish from the stock population (n = 20; week 0). The fish were culled using a Schedule 1 approved method (overdose of MS-222 at 0.05% (w/v) followed by confirmation of death), and frozen on ice, transported back to laboratory (Institute of Aquaculture, University of Stirling) and stored at − 80 C for whole body cortisol analysis (protocol described below).

At the end of the experiment, we sampled fish from each tank prior to, and following an acute stress event. Firstly, we sampled fish from each tank (n = 10 per tank; week 12), following an overdose with MS222, blood samples were taken for plasma cortisol analysis. By week 12, fish had grown (mean 30.82 ± 4.88 g), facilitating the extraction of blood samples, allowing for a more robust and clean measure of cortisol than whole body cortisol^74^. A heparinised syringe withdrew approx. 2 ml of blood (dependant on the size of individual fish) from the caudal vein, which was then chilled on ice. Blood samples were centrifuged for 10 min at 3500×*g*, following which plasma was transferred and frozen on dry ice. At this stage, brain tissue was also extracted for molecular analysis of RNA gene expression. Samples were immediately frozen on dry ice and stored at − 80 °C until analysis (details below). One week later (week 13), fish were exposed to an acute stress event (mild stressor: chasing with a net for 5 min). 30 min following the acute stress event, fish were sampled again (n = 10 per tank) and blood and brain tissue was extracted.

#### Blood plasma and whole-body cortisol analysis

Plasma cortisol was extracted by LLE (liquid–liquid phase extraction). Briefly, 2 ml of ethyl acetate was added to both samples and calibrants, then 500 µl potassium chloride solution (0.88% w/v). Samples were mixed and centrifuged at 14,000 rpm for 5 min. The supernatant was separated, with a second ethyl acetate extraction (500 µl) performed on the remaining lower phase. Pooled supernatant was dried under a stream of nitrogen and resuspended in 100 µl of cold 1:1 (v/v) methanol to water solution. After mixing, samples were centrifuged at 14,000 rpm for 2 min.

Whole body cortisol samples were homogenised in 15 mL water using an Ultra Turrax. 500 µl of methyl-tert-butyl ether (MTBE) and 500 µl of 1% (w/v) potassium chloride solution added to 250 µl of homogenate, then mixed, centrifuged at 14,000 rpm for 2 min, and supernatant isolated. A second MTBE extraction (500 µl) was performed, pooled with the first, and the extracts dried under nitrogen. Samples were resuspended in 60 µl of 1:1 (v/v) methanol/water solution and centrifuged at 14,000 rpm for 2 min. d4-cortisol was used as the internal standard in both approaches.

Cortisol was analysed using LC–MS/MS (Acquity I-Class UPLC coupled to a Waters Xevo TQS mass spectrometer). Chromatographic separation was achieved with an Acquity HSS T3 (Waters) 2.1 × 50 mm column (1.8 µm) with a 5 mm guard column. The mobile phase comprised a binary solvent system: MilliQ water (solvent A) and methanol (solvent B), both containing 0.1% (w/v) ammonium formate with 0.1% (v/v) formic acid. 12 µl of sample were injected into the column. The instrument was operated in positive electrospray ionization (ESI) mode using MassLynx V4.1 software (Waters). Limits of detection and quantification of 0.02 and 0.06 ng/ml, respectively, were achieved with sample inter-assay CV (n = 5) = 7.1% and intra-assay average %CV (n = 9) = 1.1%.

### Molecular analysis

#### Primer design and cloning

Primers (Supplemental Table 1) were searched from literature where available or designed with a primer design tool (https://www.ncbi.nlm.nih.gov/tools/primer-blast/). All primers were found specific for all target genes after the blasting process (http://www.ncbi.nlm.nih.gov/BLAST/). Touchdown PCR protocol was run to amplify the cDNA product in each of the target genes from a pull of brain samples. The amplified PCR product was verified in a 2% agarose gel and an aliquot was collected and purified by a commercial kit (Quiagen). Fragments were cloned using pGEM-T easy vector and JM109 competent cells (Promega). Plasmid DNA was obtained using QIAprep Spin Miniprep Kit (Quiagen) and sequenced to verify their identity (Eurofins genomics Europe). The concentration of the product was measured with a spectrophotometer (Nanodrop ND 2000C). The number of copies/μg RNA was calculated by using the molecular weight of the product and Avogadro’s constant. The plasmid solution was serially diluted to obtain a standard series from 10^7^ to 10 copies/μl.

#### RNA isolation and absolute qPCR quantification

Total RNA was extracted from whole brains by using the TRIZOL-reagent method (EE and NE pre-stressor n = 12, EE and NE post-stressor n = 24). 2 µg of total RNA was used to obtain cDNA, using (RT2 First Strand Kit, Quiagen). Transcript copies of target genes were determined by real-time quantitative PCR (qPCR) using a Biometra TOptical thermocycler. Analyses were performed on 1 μl of diluted cDNA (1/20) using the Luminaris HiGreen qPCR Master Mix (ThermoFisher Scientific), containing 100 nM of each primer. Transcript levels were quantified by using standard curves which were run under the same conditions.

#### Brain 5HT and 5HIAA analysis

The whole brains (n = 24 for each treatment) were homogenized using ultrasonic disruption in 300µL of pre-cooled mobile phase buffer (63.9 mM NaH2PO4, 0.1 mM Na2EDTA, 0.80 mM sodium 1-octane sulphonate, and 13% (v/v) methanol, pH adjusted to 2.95). After homogenization the samples were centrifuged (16000 g, 10 min) and the supernatants were diluted (1:5) with the mobile phase. 20 µl were injected into the HPLC system, which consists of a Jasco PU-2080 pump, a 5 µm analytical column (Phenomenex, Nucleosil C18, 150 mm length × 4.6 mm diameter) and an ESA Coulochem II detector. The oxidative potential was set with a double analytical cell (M5011) at + 40 mV in the first electrode and + 340 mV in the second one. The concentrations of 5-hydroxytryptamine (5HT) and 5-hydroxyindoleacetic acid (5HIAA) were quantified according to^75^. Data were integrated using ChromNAV version 1.12 software (Jasco Corp.) and relativized against total protein concentration, which was estimated using the bicinchoninic acid method^76^. The detection limits for 5HT and 5HIAA were 0.4 and 1.3 pg, respectively, per injection (20µL), with a signal-to-noise ratio of 3.

### Statistical analysis

Data analysis of morphological and behavioural traits was carried out using R software (version 4.3.0)^77^. Model assumptions were visualised with the “ggplot2” package^78^. Physiological traits were analysed, and all graphs were created with GraphPad Prism 8.0.1. Mixed model structure and selection was based on either Akaike Information Criterion (AIC) or Bayesian Information Criterion (BIC), dependant on model frameworks. All models assumed Gaussian error structures, deemed acceptable based on visual inspection of the model residuals. In statistical tests, levels of significance were set at p£0.05 for all statistical tests.

- Morphological

A linear mixed effect model (LMM) (lme4 package;^79^), was used to analyse the effect of enrichment on fish growth by measuring the length and weight of fish, comparing these traits at the beginning to the end of the experimental treatment. The standard assumption being that random effects and residuals are normally distributed. Both response variables (*length* and *weight*) were modelled independently and used identical fixed effect structures of *treatment* (factor with 2 levels: EE or NE treatment groups), and *session* (factor with 2 levels: week 0 and week 12). We also included *tank* as a random effect to control for repeated measures on each tank.

To analyse the effect of the enrichment on the morphological traits *dorsal fin damage* and *body condition* (both measured on a 1–3 scale), we used a generalised linear mixed model (GLMM), fit with MCMCglmm^80^. Both response variables (*dorsal fin damage* and *body condition*) were modelled independently with ordinal distributions, with fixed effects of *treatment* and *session*, and a random effect of *tank.* GLMM models implemented with the MCMCglmm package function under Bayesian frameworks, contrasting with frequentist models. In this Bayesian context, model significance is determined by whether credible intervals straddle zero or not, rather than relying on p-values. Nonetheless, it is possible to derive p-values using the MCMCglmm package, which represent the likelihood of observing the given data assuming the null hypothesis (that there is no effect) holds true.

- Behavioural

To measure the change in enrichment occupation across the experimental procedure, we firstly transformed the data as the variable *enrichment occupation* was measured on a percentage scale. The data was converted to proportions, then square-root transformed to conform to normality^81^. We used a LMM (lme4 package;^79^), with a fixed effect structure of *week* (a continuous variable, from 0 to 12) and *time* (factor with 4 levels; 12 am, 6 am, 12 pm, 6 pm). We also included *tank* as a random effect to control for repeated measures from each tank, allowing individual slopes for each tank across weeks (1 + *week:tank*).

To analyse the effect of enrichment on the behavioural traits *activity,* the variable was firstly mean centred and scaled to standard deviation units to ease interpretation of results^82^. We used a LMM with a fixed effect of *treatment* (a factor with 2 levels; EE and NE), with further fixed effects of *week* and *time,* and *tank* as a random effect*.* For *group cohesion* (measured on a 0–2 scale, 0 = tight cohesion, 1 = loose cohesion, 2 = dispersed), we used a GLMM, fit with MCMCglmm with an ordinal distribution. We included fixed effects of *treatment* and *session*, with an interaction term (*treatment:session*) to measure any relationship between the two, and a random effect of *tank.* To analyse the effect of the enrichment on the behavioural response during the novel object tests, firstly we transformed the variable *latency* (*to resume normal activity*) using square root transformation, so that the raw data was more normally distributed^81^. We then fit a LMM with *latency* as the response variable, fixed effects of *treatment* and *trial* (a factor with 6 levels, representing each novel object trial), and a random effect of *tank.*

- Physiological

### Cortisol analysis

To measure the effect of enrichment on the stress response, we analysed the cortisol data for each treatment, before and after exposure to the stressor event (chasing with a net). Firstly, the population base line measure of whole-body cortisol was compared between both treatment groups using a t-test. Second, to measure any effect of the enrichment treatment on cortisol expression pre and post stressor at the end of the experiment, we used an LMM. The variable *cortisol* (blood plasma cortisol) was square root transformed to better fit normality^81^, then a LMM was fit with *cortisol* as the response variable, fixed effects of *treatment* and *session* (a factor with 2 levels: pre- and post-stressor), and a random effect of *tank.*

### Monoamines and brain gene expression

To measure the effect of the enrichment on monoamines and brain gene expression, we analysed the monoamine concentration and absolute expression of the RNA transcripts for each treatment (treatment groups pooled together for analysis), before and after exposure to the stressor event. A two-way ANOVA followed by multiple variable Tukey’s t-test were performed to evaluate any effect of the enrichment treatment on monoamines and gene expression, using categorical variables *treatment* and *session.*

## Results

### Mortality

Survival by the end of the 12-week experimental period (excluding all fish removed from the tanks during sampling) was 98.6% for the enriched treatment group (n = 5 mortalities), and 97.6% for the non-enriched treatment group (n = 9 mortalities).

### Morphological

No effect of the enrichment on any morphological trait was observed. No difference was found in length between the NE group (mean ± SE = 14.07 ± 0.167 cm), and the EE group (13.96 ± 0.167 cm; t = 0.554, df = 8, *p* = 0.595; Supplementary Fig. S1). Similarly, no difference in weight was found between treatment groups (NE = 31.03 ± 0.742 g; EE = 30.62 ± 0.742 g; t = 0.462, df = 8, *p* = 0.656) meaning all animals grow under a similar pattern and were homogenously distributed in weight and length at the end of the trial.

The GLMMs did not provide support for an effect of the enrichment on *dorsal fin damage* or *body condition.* For *dorsal fin damage,* the estimated effect of the enrichment treatment was non-significant, with confidence intervals (CI) straddling zero (posterior mean = − 9.099, 95% CI − 23.761 to 5.056, pMCMC = 0.092; Supplementary Fig. S2). A similar non-significant effect of treatment is observed for *body condition* (− 1.146, 95% CI − 8.71 to 3.00, pMCMC = 0.922; Supplementary Fig. S2). These findings suggest that the experimental conditions did not result in adverse effects on dorsal fin damage or body condition, which are indicators of potential aggression or disease. However, the potential for high individual variability (as seen by the broad CIs) and a relatively small sample size may have limited the ability to detect subtle effects. Nonetheless, the consistency of results across tanks enhances confidence in the robustness of these conclusions.

### Behavioural

A high percentage of fish were observed under the enrichment in the first 2 weeks of the experiment (week 1; mean ± SE = 47.9% ± 0.757, week 2; 41.8% ± 0.738; Supplementary Fig. S3A). Enrichment occupation declined each week, and a significant effect of time was observed (− 0.133 ± 0.021, t = − 6.425, df = 4 *p* = 0.003; Supplementary Fig. S3B). Of note, a strong significant decline in enrichment occupation was observed at 12 pm (− 0.247 ± 0.057, t = − 4.334, df = 1.144, *p* < 0.001), corresponding to the timing of daily husbandry routines. These coefficients are on the square root scale, and caution is advised with their interpretation. However, the sign and strength of the relationship is meaningful and relevant here.

Activity declined significantly over time across both treatments (− 0.056 ± 0.004, t = − 13.954, df = 4787, *p* < 0.001), but no effect of treatment was observed (− 0.005 ± 0.003, t = − 1.743, df = 4787, *p* = 0.081; Supplementary Fig. S4). Following exposure to the novel object test, fish in the EE group were faster to resume normal swimming compared to NE fish (4.304 ± 1.124, t = 3.830, df = 18.182, *p* < 0.005; Supplementary Fig. 5A). This relationship was also observed over time (3.069 ± 0.392, t = 7.830, df = 9.232, *p* < 0.001) with EE fish displaying higher resilience to novelty.

**Fig. 5:**
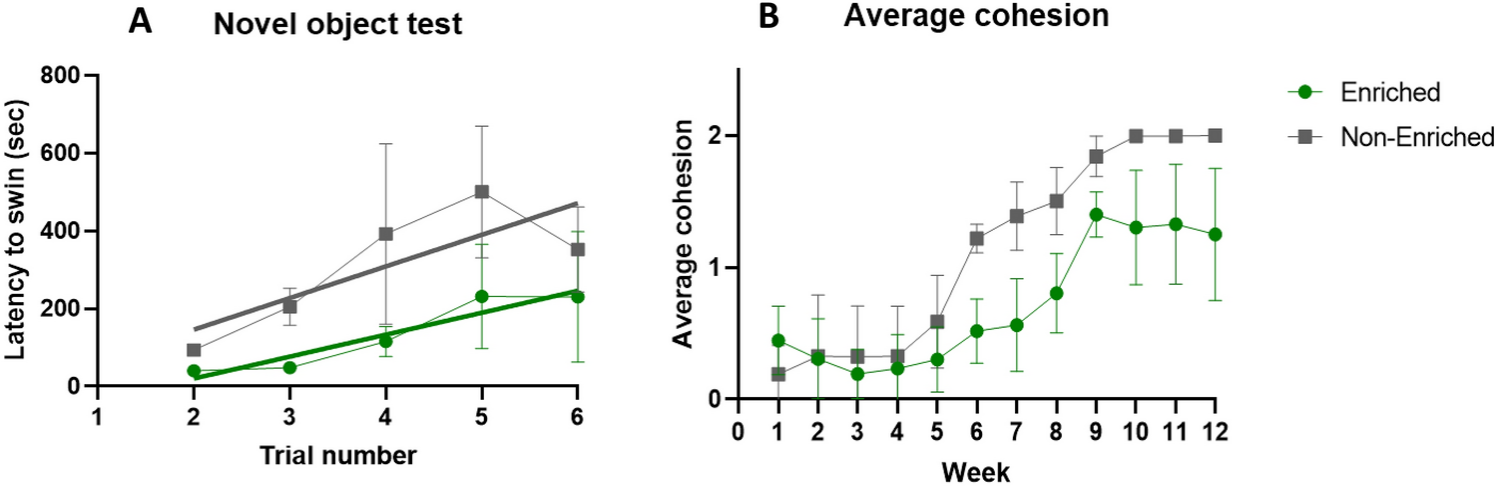
(**A**) Latency to resume normal swimming following presentation of novel object (camera). Data are expressed as means (± SD), fitted with regression lines, showing EE tanks (green points) and NE tanks (grey points). (**B**) Average cohesion profiles (0 = tight cohesion, 1 = loose, 2 = dispersed) measured across 12 weeks for EE and NE tanks.

A significant interaction between treatment and time on group cohesion was observed (treatment:week = 9.802, 95% CIs 2.119 to 18.905, pMCMC < 0.001), indicating cohesion differs between treatment groups over time, with EE fish displaying loose cohesive profiles over time, and NE fish becoming more dispersed). EE fish display greater variability in cohesion compared to NE fish (residual variance estimate: post.mean = 822.5, 95% CIs 56.47, 2666), with variability becoming more pronounced in the latter weeks of the experiment (Fig. 5B). Although there was a non-significant effect of enrichment on group cohesion (− 29.579, 95% CI − 82.084 to 16.007, pMCMC = 0.192), this estimate relates to group cohesion at week 1 only (the intercept), and interpretation of the interaction term is more relevant here. Of note, there was a significant positive effect of time on group cohesion (week = 8.334, 95% CI 2.719 to 16.829, pMCMC < 0.001). This relationship can be visualised in Fig. 5B for clarity, all fish display ‘tight’ cohesion at the beginning of the experiment, becoming more dispersed over time in both treatment groups.

### Physiological

No difference was observed in baseline cortisol expression between NE (636.578 ± 346.762 pg.g) and EE treatments (629.3 ± 464.7 pg.g; t = 0.828, df = 42.73, *p* value = 0.412; Supplementary Fig. S5B). A significant effect of acute stress was observed with plasma cortisol increasing across both treatments following acute stress (26.23 ± 5.71, t = 4.179, df = 168, *p* < 0.001). No effect of the EE treatment was observed (− 0.453 ± 0.469, t = − 0.965, df = 8, *p* = 0.363; Supplementary Fig. S5B). Of the total samples analysed, 3.1% were below the detection limit. In these cases, the limit value detectable has been used for statistical purposes to ensure accuracy and reliability.

**Fig. 6:**
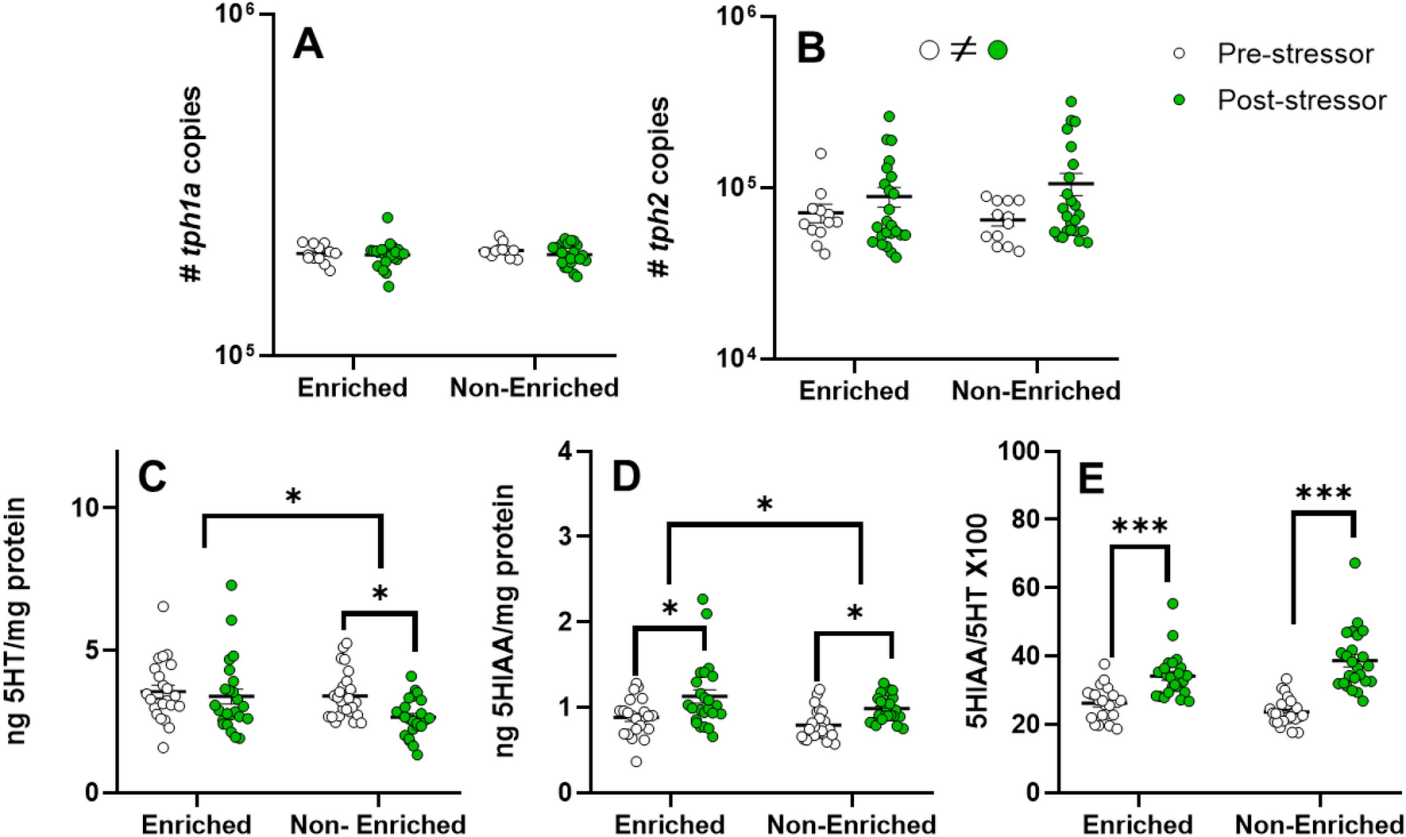
(**A**) Expression levels of tph1a (tryptophan hydroxylase 1a), (**B**) tph2 (tryptophan hydroxylase 2), (**C**) tissue content of 5HT (5Hydroxytriptamine, serotonin), (**D**) 5HIAA (5hydroxyindolacetic acid) and (**E**) ratio between 5HIAA and 5HT in the brain of S. Salar reared during 12 weeks with or without structural environmental enrichment (enriched vs non-enriched) before stress, white dots, and after exposure to a stress event (5 min net chasing), green dots. Data are expressed as mean (± SEM). Significant differences among groups are indicated with * or ≠ (* p* < 0.05), **(*p* < 0.01) and ***(*p* < 0.001).

Figure 6 shows the brain concentration of 5HT, 5HIAA, the ratio (5HIAA/5HT) and mRNA abundance of serotonin synthesis rate-limiting enzymes *tph1* and *tph2*. The highest levels of 5HT and 5HIAA were found in EE fish (F_1,93_ = 4.981; *p* < 0.05 and F_1,93_ = 5.274; *p* < 0.05, respectively). In addition, our results indicate that the acute stressor induced significant increases in 5HIAA levels (F_1,93_ = 18.14; *p* < 0.0001), and in the 5-HIAA/5-HT ratio (F_1,93_ = 77.34; *p* < 0.0001), showing a significant interaction between the enrichment condition (F_1,93_ = 4.493; *p* < 0.05). However, the stressor only significantly impacted NE fish in 5HT (q = 3.779, df = 93 *p* = 0.0434), which shows lower amine levels. The detection limits for 5HT and its metabolite 5HIAA are significantly below the values obtained in the analyses of this experiment. The minimum quantified value for 5HT was 27.33 pg in 20µL of injection, while its detection limit was 0.4 pg. For 5HIAA, the minimum value obtained was 6.58 pg, with a detection limit of 1.3 pg, indicating that the lower readings for both analytes are accurate and differentiable. Results of gene expression related to serotonin synthesis show no significant change in tph1a transcript levels (Fig. 6A). However, expression of *tph2* (Fig. 6B) was significantly modulated by the stressor (F_1,68_ = 4.037; *p* < 0.05) independent of treatment groups.

**Fig. 7:**
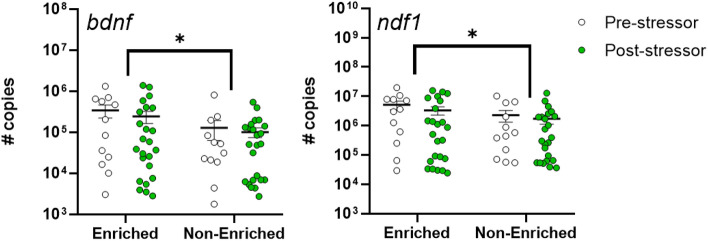
Changes in mRNA abundance of bdnf (brain-derived neurotrophic factor), ndf1 (Neurogenic Differentiation Factor 1) in brain of S. Salar reared during 12 weeks with or without structural environmental enrichment (enriched vs non-enriched) before stress, white dots, and after exposure to a stress event (5 min net chasing), green dots. Data are expressed as means (± SEM). Significant differences among groups are indicated with *(*p* < 0.05).

In terms of the expression of genes related to cognition (Fig. 7) and stress response (Supplementary Fig. S6), a significant increase was found in *bndf* and *ndf1* transcript levels in EE fish (*bndf*: F_1,68_ = 5.379, *p* < 0.05, *ndf1*: F_1,68_ = 4.250, *p* < 0.05), meanwhile there was no significant effect of acute stress on these genes (F_1,68_ = 0.6689, *p* = 0.416 and F_1,68_ = 1.211 *p* = 0.275). Conversely, acute stress promotes a significant increase in expression of *hsp90* (F_1,68_ = 4.556, *p* < 0.05) and *cfos* (F_1,68_ = 4.300, *p* < 0.05), without detected enrichment effect (F_1,68_ = 0.512, *p* = 0.477, and F_1,68_ = 0.024, *p* = 0.877 respectively; Supplementary Fig. S6). No significant difference was found between treatment groups in terms of transcript levels of genes related to HPI axis (*crfb*, *gr1*, and *gr2*).

Correlation analysis (Supplementary Fig. S7) indicates positive consistency between the levels of the ndf1 and *bdnf* (*p* < 0.001, Pearson index = 0.708), and between *ndf1* vs *hsp90* (*p* < 0.05, Pearson index = 0.257), and *bdnf* vs *hsp90* (*p* < 0.05, Pearson index = 0.308). In particular, in these last two pairs, there was a high correlation between fish reared in the enriched treatment (*p* < 0.001, Pearson Index = 0.609 and 0.728 respectively), which was not present in the non-enriched treatment group (*p* = 0.270, Pearson Index = 0.189 and *p* = 0.120, Pearson Index = 0.264, respectively).

## Discussion

In this study we sought to examine whether rearing early life *S. salar* in enriched housing conditions, compared to barren environments, can improve the welfare of this species when housed in captivity for research purposes. The results obtained here provide support for beneficial effects of enriched rearing environments on welfare in this species. Firstly, and of considerable importance, the provision of EE did not negatively affect growth or result in a higher degree of fin damage, compared to NE fish. EE significantly affects fish behaviour, specifically social behaviour, changing group cohesion over time. Furthermore, EE accelerates recovery from exposure to novel objects, producing less neophobic (fearful) behavioural profiles. Concurrently, EE promotes cognitive capacity and neurogenesis at the physiological level, as evidenced by the increased expression of related genes. This augmentation in cognitive function and neurogenesis correlates with an enhanced ability to respond to stress, reflected in higher serotonergic activity. Taken together, these findings suggests that the presence of EE improves the well-being of salmon parr in captivity when exposed at the early life stages, enhancing stress-coping behavioural profiles and cognitive abilities in line with an improved mental state. In what follows, we discuss the implications of our findings, and their importance in the wider context of fish welfare research.

The presence of EE, here in the form of suspended artificial plants, significantly influenced salmon parr behaviour. Both EE and NE fish exhibited tight group cohesion during early life stages, reflecting a natural schooling tendency for predator defence^83^. As fish matured, NE fish showed more dispersed spatial profiles compared to EE fish, potentially indicating increased exploratory behaviour in the absence of enrichment (although exploration was not measured directly here). In contrast, on average EE fish maintained loosely cohesive group dynamics. These findings challenge expectations, as enrichment has been associated with increased dispersion and exploration^84^, due to reduced anxiety^85^, which can promote positive welfare^46^. NE fish’s dispersed profiles may result from enhanced exploratory tendencies, or from the absence of clumping around enrichment structures, leading to a more even spatial distribution. Concurrently, the structural complexity in EE tanks may encourage exploration in addition to aggregation under the enrichment, promoting the loose spatial cohesion observed here. To clarify this relationship further, a detailed analysis targeting the effects of EE on group cohesion and exploration behaviour would be beneficial. Furthermore, EE fish exhibited greater variability in cohesive behaviour compared to NE fish, suggesting behavioural flexibility is fostered by environmental complexity, an effect observed in other fish species^22,27^. Behavioural flexibility supports diverse social interactions and promotes natural behaviours, aligning with the enrichment goal of enhancing positive welfare states^16,46,86^.

EE can also influence social behaviour in group-housed animals by reducing the opportunity for aggressive interactions (for a review see^12^;). Despite salmon parr’s territorial nature, it has been suggested that EE can stabilise these dynamics by offering refuge and reducing visual contact among potential rivals^40,87,88^. We see low levels of fin damage or differences in body condition across both treatment groups, suggesting EE has no effect on aggression here, contrary to findings from similar studies^46,69,89^. This may be a result of suitable life-stage stocking densities used here, paired with a predictable food source, which has been shown to reduce aggression^90,91^, as opposed to the provision of EE. Any effect of EE on group dynamics and aggression may become more apparent as fish develop and tank space becomes more limited^40^. This highlights an important area for future research investigating the effects of EE provision and stocking densities on aggression at different life stages.

Furthermore, we find EE fish are quicker to recover following exposure to a novel object compared to NE fish. High risk taking and less fearful responses when exposed to novelty are behavioural traits characteristic of proactive stress coping styles^29,92,93^, as opposed to fearful risk-aversive reactive coping styles. Provision of EE here may be promoting stress coping profiles better adapted to captivity, such as reduced anxiety and fear, as commonly observed in terrestrial animal models^94–96^, and other aquaculture species^97,98^. For example, captive European seabass (*Dicentrarchus labrax*) provided with vertically suspended enrichment, promoted proactive behaviour and behavioural flexibility during a risk-taking task, compared to control fish reared without enrichment^97^. Behavioural flexibility is linked to cognitive capacity and contributes to information processing, enabling animals to respond flexibly to novel experiences and changing environmental conditions^22,93^. This is an adaptive trait that can enhance welfare in captive animals^99^, and provision of EE can also promote behavioural flexibility and cognitive capacity, as previously observed in juvenile salmon^21^.

EE is considered a modulator of cognitive capacity in captive fish^40^. Neurogenesis is suggested to be directly related to environmental complexity, with higher environmental complexity correlated to higher neuronal growth, which translates into enhanced cognitive capacity^21,100,101^. Several occurrences are known, for example behaviours such as foraging for prey, predator avoidance, and spatial learning improve when animals are reared in complex environments^21,22,102^. Our findings align with previous studies, as the environmental complexity provided by EE here results in increased expression of neurogenesis markers *bdnf* and *ndf1*. Both factors have been linked to neurogenesis, neuronal maintenance, neuronal survival, plasticity and neurotransmitter regulation in fish^103^. In addition, evidence from mammalian studies suggests a close relationship between transcription factor ndf1 and the stimulation of nerve growth through the bdnf/trkb pathway^104^. In our experimental conditions, we observe fish reared with EE show higher levels of *bdnf* and *ndf1*, suggesting increased neuronal proliferation in these individuals. This finding translates to higher cognitive capacity in EE housed fish^64^ Indeed, the high correlation shown between the expression of both genes supports the interaction proposed above.

However, the positive effects on cognitive ability derived from EE are not permanent and are highly dependent on exposure to stress. In zebrafish (*Danio rerio*), EE derived neurogenesis has been shown to reduce after stress^105^. In juvenile salmon, EE promotes neuronal growth, which is rapidly reversed when EE is withdrawn^106^. In Trinidadian guppies (*Poecilia reticulata*), even maternal exposure to intermittent stress has been found to negatively affect the cognitive capacity of their offspring^107^. In contrast, our data do not show a negative effect of acute stress on the expression of neurogenesis-related factors, in either EE or NE fish. Furthermore, we did not observe a significant effect of stress on the expression of genes related to the HPI axis. However, we did detect activation of the cellular response to stress, evidenced by higher levels of hsp90 and cfos, which could indicate a neuronal integration of signals related to stress events. Considerable evidence indicates that a coordinated hsp70/hsp90 interaction enhances the binding capacity of glucocorticoid receptors, increasing their sensitivity and affinity to stress-related hormones^108^, with cortisol (the main stress-related hormone) having been shown in teleost fish to promote hsp70 binding to the glucocorticoid receptor^109^. In this regard, we found a significant correlation between neurogenesis and stress response-related cellular genes in EE reared fish, suggesting that fish exhibiting higher cognitive abilities also develop a high level of *hsp90*. This finding may be indicative of an increased sensitivity to stress events, thus indicating a superior competence of these fish to integrate stress-related stimuli as a stress-coping mechanism. This finding suggests the provision of EE in research is essential (and notably for cognitive research) to improve experimental validity (and hence replicability), as recommended in other animal models^110^.

In terms of cortisol levels, the major hormone related to the stress response^62^, there does not appear to be an effect of EE per se, as our data suggest that cortisol levels are similar at baseline and following acute stress exposure in both treatment groups, as previously observed^111^. In our experimental protocol, acute stress was applied at the end of the experiment, and cortisol levels were assessed before and 30 min after the application of the stressor. Plasma cortisol levels have been found to increase significantly in fish exposed to the stressor, suggesting an activation of the hypothalamic-pituitary-interrenal axis and the expected subsequent release of glucocorticoids into the bloodstream, a phenomenon widely reported in several fish species (reviewed by^112^). This increase in cortisol levels is significant both in the presence and absence of environmental enrichment. However, the degree of significance and magnitude of this increase is more marked in fish reared in enriched environments, which is consistent with previously described results on gene expression and suggests a possible increased sensitivity of these animals to stressful events, thus implying a higher cognitive capacity.

In this line, previous studies have pointed out that fish reared with different kinds of structural enrichment display high cortisol levels after exposure to stress^113,114^. However, some studies indicate different relationships i.e. the presence of enrichment reduces cortisol magnitude after stress^115^, or no differences was found between rearing fish with or without enrichment in salmon^89^. Furthermore, previous studies suggest that the presence of EE not only generates a differential sensitivity to the stressor, but also modifies the dynamics of cortisol release, with the presence of EE being associated with a quick recovery of basal cortisol levels^60,115^ consistent with a greater ability to recover from the threat. Therefore, the discrepancies among fish species, enrichment type, stressor model, rearing conditions, and experimental designs, indicate that the stress response and the effect of enrichment on its regulations is complex and standardized research is needed to elucidate the direction and strength of the effect.

Previous studies have indicated that stressful stimuli activate the serotonergic system in teleost fish^63^, a result similar to that observed in our study, where we found greater serotonergic activity at the CNS level in fish subjected to chase stress. This statement comes from a higher degradation of 5HT in its major oxidative metabolite 5HIAA, resulting in a higher activity ratio and conversely, a higher expression of the limiting enzyme in the synthesis of serotonin tph2 located at the hindbrain and diencephalon level^116^. Here, fish reared with EE show higher levels of 5HT and 5HIAA, indicative of a higher basal turnover, in line with previous observations in *Gobiocypris rarus*^117^. Moreover, fish reared with EE do not show a decrease in 5HT content after stress, indicative of a higher 5HT turnover capacity, similar results to those obtained in fish subjected to high stocking density^34^. It has been previously described that low levels of 5HT could be indicative of behaviours compatible with stages of anxiety or depression^118,119^, similar to those observed in mammals^120^. In our findings, stress does not reduce 5HT levels in SEE fish, which can be interpreted as increased resilience of these animals, a higher stability of 5HT levels may translate into a lower predisposition to fall into stages of anxiety or depression. Furthermore, deficiencies in the serotonergic system are associated with impaired cognition, as 5HT is closely related to neurogenesis in fish^121^, so this increased resilience at the level of 5HT content could potentially have a beneficial effect on the cognitive abilities of salmon. However, caution is required when using environmental enrichment, as its effects are highly dependent on factors such as fish species, developmental stage, and the type and abundance of enrichment provided. For example, in the case of gilthead seabream (*Sparus aurata*), the addition of substrate to tanks reduces serotoninergic activity^34^, while introducing complex environments with ropes enhances this activity in adults^33^. However, such enrichment has no effect on juveniles^39^.

Here, we provide support for beneficial effects of enriched early life rearing environments on the welfare of *S. salar* when housed in captivity. In particular, we show EE affects social behaviour, changing group cohesion over time, producing less reactive and neophobic behavioural profiles. Furthermore, EE promotes cognitive capacity and neurogenesis at the physiological level, indicating an enhanced ability to respond to stress, reflected in higher serotonergic activity. Taken together, these findings suggests that the presence of EE improves the well-being of early-life salmon in captivity, and potentially in later life, highlighting an important area for future research investigating the effects of EE provision on well-being at different life stages. Our findings also suggest that the provision of EE enhances resilience in early-life salmon, in addition to stress-coping behavioural profiles and cognitive abilities in line with improved mental states indicative of animals experiencing positive welfare.

## Supplementary Information

Supplementary Information.

## Data Availability

We verify that all information can be found in the manuscript or in the supplementary information.
