# Supplementary material for: Early-life environmental enrichment promotes positive animal welfare for juvenile Atlantic salmon (Salmo salar) in aquaculture research
Source: Sci Rep. 2025 Feb 18;15:5828. doi: 10.1038/s41598-025-88780-0 (PMC11836395; doi:10.1038/s41598-025-88780-0)
Supplement: Supplementary file 1 — Supplementary Information. [file 41598_2025_88780_MOESM1_ESM.docx]

**Supplementary Information**

*Supplementary Table S1: Nucleotide sequences of primers used to evaluate absolute expression of target transcript by RT-qPCR*

|  | | Forward primer | Reverse Primer | Amp | Reference |
| --- | --- | --- | --- | --- | --- |
| Brain-derived neurotrophic factor | *bdnf* | ATGTCTGGGCAGACCGTTAC | GTTGTCCTGCATTGGGAGTT | 158 | XM_014175919.2 |
| Neurogenic differentiation factor 1 | *ndf1* | CAATGGACAGCTCCCACATCT | CCAGCGCACTTCCGTATGA | 224 | NM_001139808.1 |
| Synaptotagmin-17 | *syt* | GTTCAGCTTCAGAGTCCCCC | GGAGAACTGGCCAATCACGA | 121 | XM_045715888.1 |
| Proliferating cell nuclear antigen | *pcna* | TGAGCTCGTCGGGTATCTCT | CTCGAAGACTAGGGCGAGTG | 224 | XM_014161524.2 |
| Proto-oncogene c-Fos | *cfos* | GGACTGGGAGCCTCTCTACA | TCCGCCTCTGGGTAGGTAAA | 126 | XM_014206157.2 |
| Corticoliberin-1 | *crfb* | TCCATCACTCGTGGAAAAGGA | CAGGGGTTCAACGAGATCTTCA | 91 | XM_014181363.2 |
| Glucocorticoid receptor 1 | *gr1* | ACGACGATGGAGCCGAAC | ATGGCTTTGAGCAGGGATAG | 107 | XM_045717356.1 |
| Glucocorticoid receptor 2 | *gr2* | TGGTGGGCTGCTGGATTTCTGC | CTCCCTGTCTCCCTCTGTCA | 204 | XM_014198678.2 |
| Heat shock protein 70 | *hsp70* | CCTGCCTACTTCAACGATTCACAGAGACA | CCAGCGATCACTCCAGCGTCCTTA | 59 | NM_001141684 |
| Heat shock protein 90 | *hsp90* | TTGCGTGGAACTAAGGTGA | CCAATGAACTGAGAGTGCT | 104 | NM_001146473.1 |
| Tryptophan Hydroxylase 1a | *tph1a* | CGACTCCATGAACATCGGGAA | AGAAGACAATCGCTGCTCGG | 134 | XM_045706353.1 |
| Tryptophan Hydroxylase 2 | *tph2* | TGAGTCACGATGTCCAAGC | TGCTCCAGCAGCTCGTTAAA | 92 | XM_014125607.2 |

*Supplementary Figure S1. Growth curve showing the mean weight of fish from two treatment groups over 12 weeks in (grams), showing both EE (green points) and NE (grey) treatment groups.*


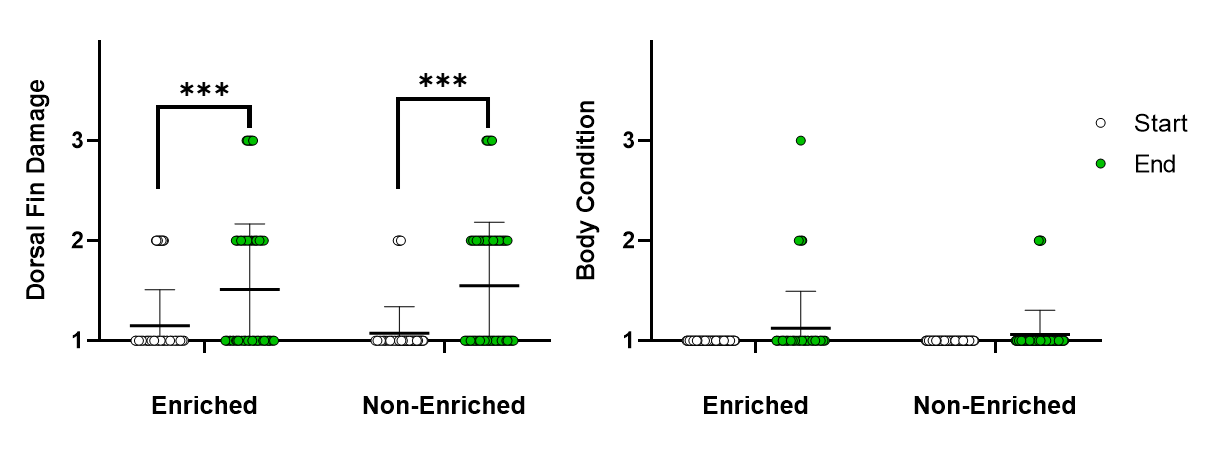


*Supplementary Figure S2. Changes in morphological welfare indicator scores of S. salar reared during 12 weeks with or without structural environmental enrichment (enriched vs non-enriched) at week 0 (start) and week 12 (end) of the experiment. Data are expressed as means (± SD). Significant differences among groups are indicated with * (p<0.05), ** (p<0.01) or *** (p<0.001). Individual plots show (A) Dorsal Fin Damage, and (B) Body Condition scores (1=normal condition, 2=minor occurrences, 3 = compromised condition; adapted from Noble et al., (2018))*

*
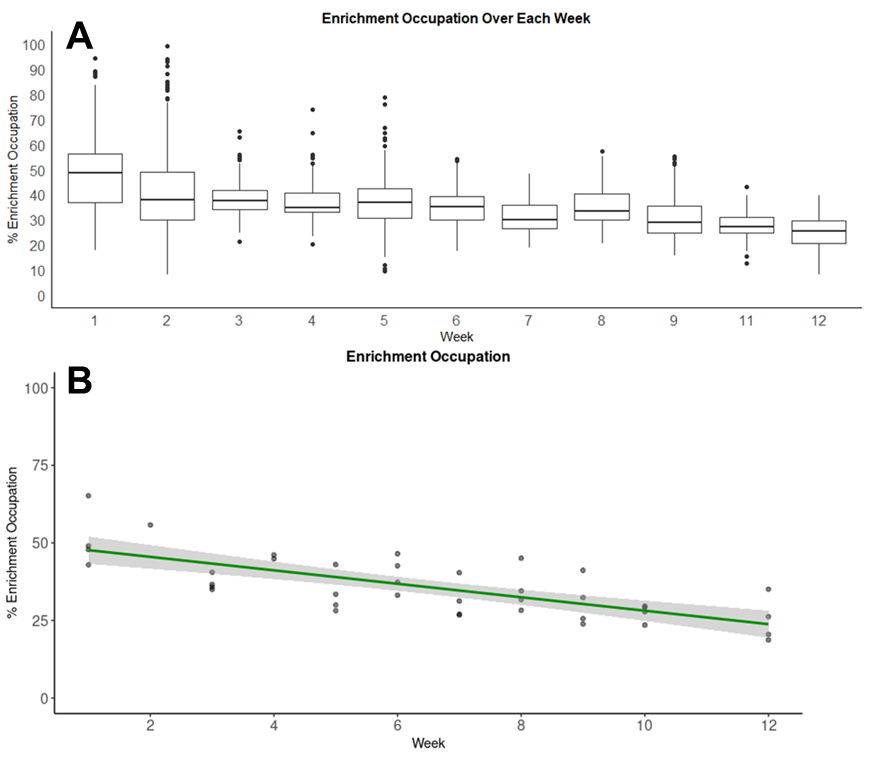
Supplementary Figure S3. Enrichment occupation across 12 weeks for enriched treatment tanks. (A) Raw data is shown as weekly boxplots of the percentage of fish in each tank residing under the enrichment structure (B) Weekly averages are shown as individual points and fitted with a linear regression line, showing 95% confidence intervals in grey.*

*
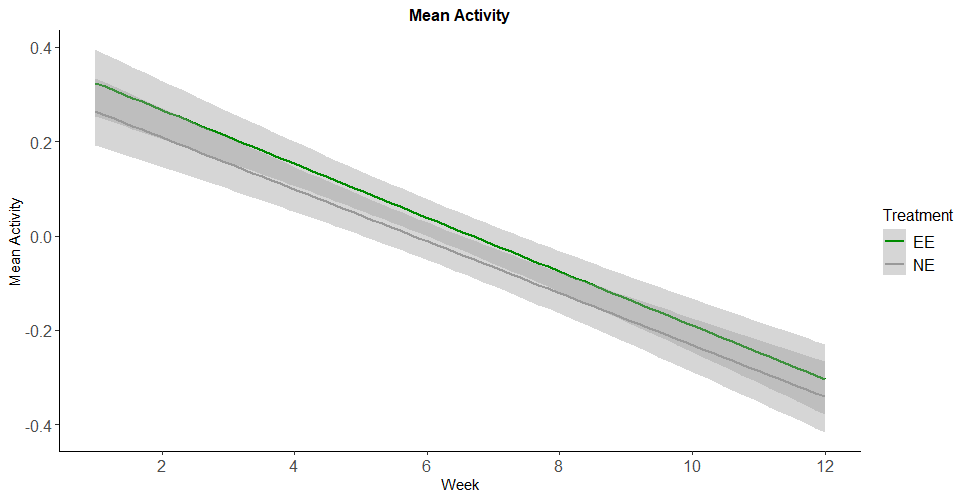
Supplementary Figure S4. Fish activity across12 weeks for both treatment groups, green lines represents enriched treatment group tanks, and grey represent non-enriched tanks, fitted with a linear regression line, showing 95% confidence intervals in grey.*


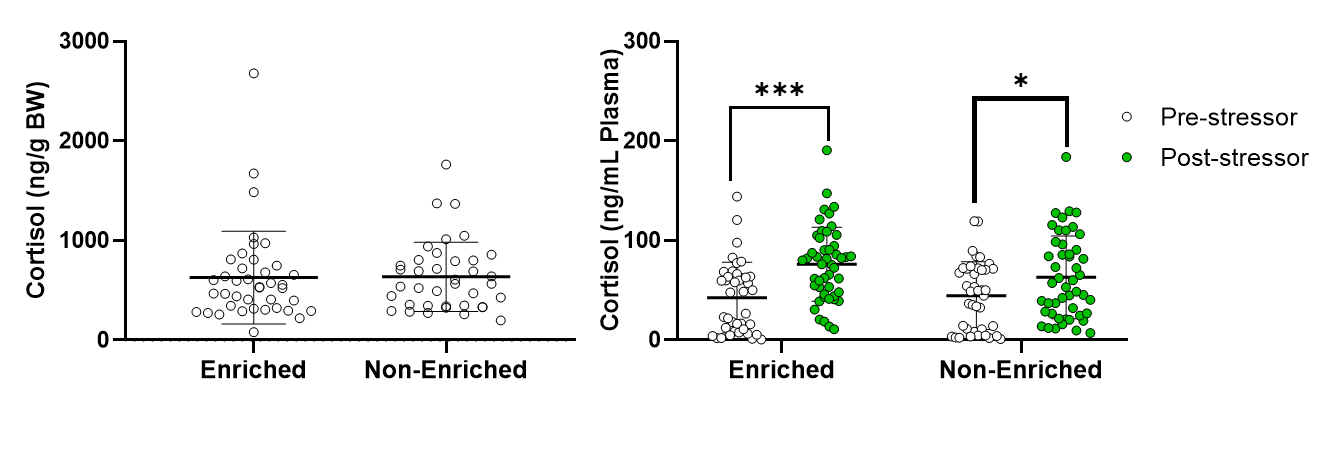


*Supplementary Figure S5 (A) Basal whole-body cortisol level of S. Salar used in this experiment. (B) Plasma cortisol levels of fish reared during 12 weeks with or without environmental enrichment (enriched vs non-enriched) pre-stress, white dots, and post-stress exposure (5 min net chasing), green dots. Data are expressed as means (± SD). Significant differences among groups are indicated with * (p<0.05) ** (p<0,01) *** (p<0,001)*


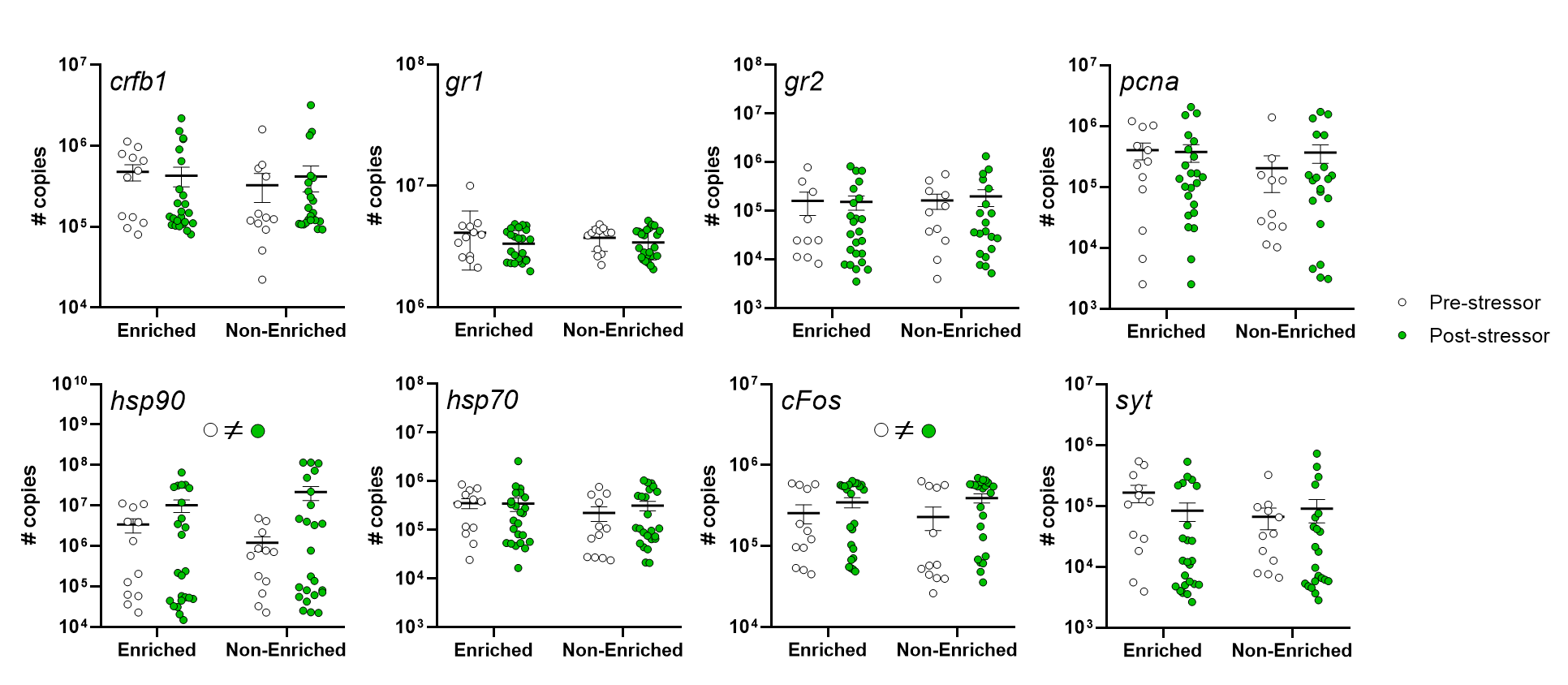


*Supplementary Figure S6. Changes in mRNA abundance of crfb1 (corticotropin releaser factor b1), gr1 (glucocorticoid receptor 1), gr2 (glucocorticoid receptor 2), pcna (Proliferating cell nuclear antigen), hsp90 (Heat shock protein 90), hsp70 (Heat shock protein 70), cFos (Fos proto-oncogene) and syt (Synaptotagmin) in brain of S. Salar reared during 12 weeks with or without structural environmental enrichment (enriched vs non-enriched) before stress, white dots, and after exposure to a stress event (5min net chasing), green dots. Date are expressed as means (± SEM). Significant differences among groups are indicated with ≠ (p<0.05)*


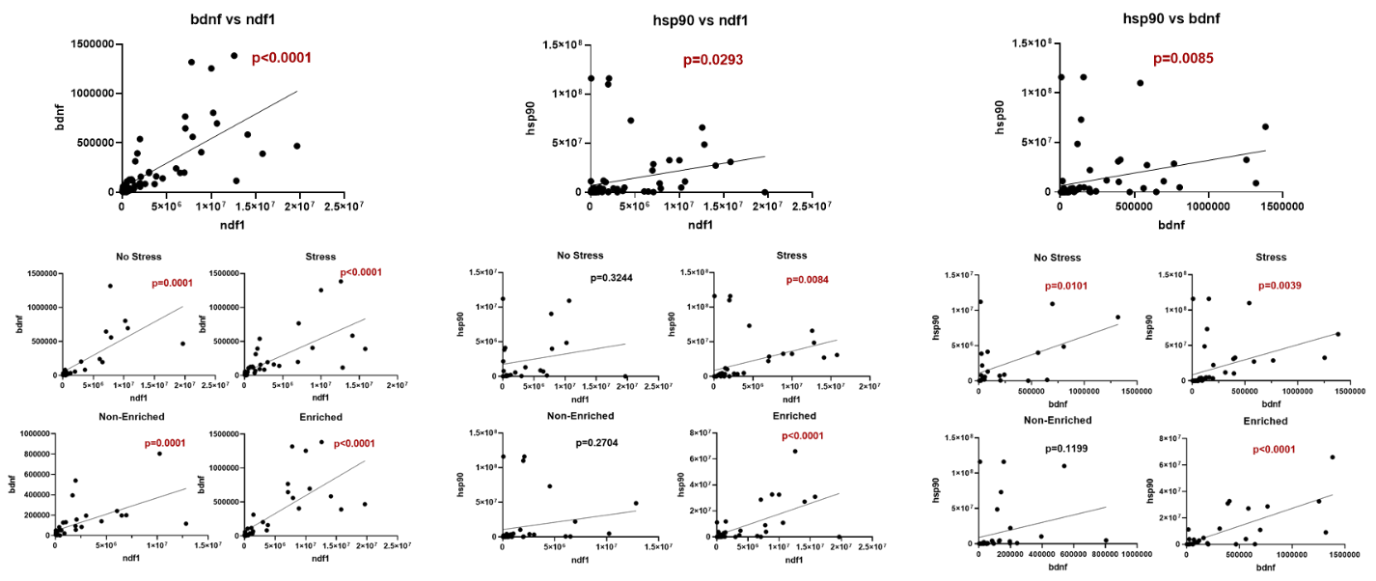


*Supplementary Figure S7. Linear regression analysis of bdnf vs ndf1, hsp90 vs ndf1 and hsp90 vs bdnf. The main plots (above) show the data as a whole, while the subplots show the data sorted by the different experimental variables. Red highlighted p values indicate a statistically significant relationship between parameters.*
